# Chemical Constituents of Essential Oil from *Lippia sidoides* Cham. (Verbenaceae) Leaves Cultivated in Hidrolândia, Goiás, Brazil

**DOI:** 10.1155/2012/363919

**Published:** 2012-02-23

**Authors:** Sandra Ribeiro de Morais, Thiago Levi Silva Oliveira, Maria Teresa Freitas Bara, Edemilson Cardoso da Conceição, Maria Helena Rezende, Pedro Henrique Ferri, José Realino de Paula

**Affiliations:** ^1^Programa de Pós-Graduação em Biologia, Instituto de Ciências Biológicas, Universidade Federal de Goiás, 74001 970 Goiânia, GO, Brazil; ^2^Instituto de Ciências da Saúde, Universidade Paulista, Campus Flamboyant, 74845 090 Goiânia, GO, Brazil; ^3^Programa de Pós-Graduação em Ciências Farmacêuticas, Laboratório de Pesquisa de Produtos Naturais, Faculdade de Farmácia, Universidade Federal de Goiás, 74605 220 Goiânia, GO, Brazil; ^4^Faculdade de Farmácia, Universidade Federal de Goiás, 74605 220 Goiânia, GO, Brazil; ^5^Instituto de Ciências Biológicas, Universidade Federal de Goiás, Campus Samambaia, 74001 970 Goiânia, GO, Brazil; ^6^Instituto de Química, Laboratório de Bioatividade Molecular, Universidade Federal de Goiás, Campus Samambaia, 74001 970 Goiânia, GO, Brazil

## Abstract

Several studies involving the family Verbenaceae, occurring in the Brazilian Cerrado, have emphasized the popular use of many aromatic species. We highlight the use of *Lippia sidoides* Cham., known as “alecrim pimenta,” native to northeastern Brazil and northern Minas Gerais. Leaves of this species were collected in antropized Brazilian Cerrado area, in Hidrolândia, Goiás, and their essential oils were extracted by hydrodistillation in a Clevenger-type apparatus and thereafter analyzed GC/MS. Among the compounds identified in this study were the most abundant oxygenated monoterpenes, followed by sesquiterpenes hydrocarbons. The oxygenated monoterpene 1,8-cineole was the major constituent followed by isoborneol and bornyl acetate. The chemical composition of essential oil described in this paper differs from that described in the literature for *L. sidoides* found in its native environment, where the major constituents are thymol and carvacrol.

## 1. Introduction

The knowledge of chemical constituents of essential oils is of fundamental importance to the pharmaceutical, food, and perfumery industries. As the use of aromatic compounds requires detailed chemical characterization and evaluation of possible modifications within their compositions, which are due to the different geographical origins and/or climatic conditions and various population genetics that can lead to the formation of different chemotypes [1, 2].

Various studies involving the Verbenaceae family have highlighted the importance of many species used within popular medicine by the presence of principle aromas [3–9]. It is worth noting that in this family, the species *Lippia sidoides*, popularly known as “alecrim-pimenta,” native to the northeastern region of Brazil and north of the state of Minas Gerais, is an aromatic species commonly used in the form of infusions and inhalations, allergic rhinitis, and in the treatment of vaginal, mouth, and throat infections [10].

Within the chemical components described of this species, thymol and carvacrol are major constituents of the essential oil [11–13], with a remarkable inhibitory activity regarding the development of microorganisms [14–17]. Moreover, the studies show variations in the concentration of thymol in different stages of the plant's development [18]. This work aims to determine the chemical composition of the essential oil of *L. sidoides *cultivated in an area of antropized cerrado in Hidrolândia, Goiás, Brazil.

## 2. Experimental

The leaves of *Lippia sidoides* Cham. were collected from three plants grown in the municipality of Hidrolândia, Goiás, Brazil (altitude 835 m, 16° 54′ 1.3′′ south, 49° 15′ 35.2′′ west) in august/2010, both northwest Minas Gerais, Brazil origin. Botanic material was identified by Dr. Marcos José da Silva, of Departamento de Biologia Geral do Instituto de Ciências Biológicas/UFG, and vouchers were deposited in the Herbarium of Universidade Federal de Goiás (UFG) under code number 45121.

Leaves were dried at room temperature and then pulverized by blade mill. Essential oil was extracted by hydrodistillation in a Clevenger-type apparatus for 2 hours from 50 g of powered leaves in 1000 mL of water. At the end of each distillation, the oils were measured in Clevenger trap, collected, dried with anhydrous Na_2_SO_4_, stored in hermetically sealed glass containers with rubber lids, covered with aluminum foil to protect the contents from light, and kept under refrigeration at −10°C until used. The essential oil was submitted to GC/MS analysis performed on Shimadzu QP5050A apparatus using a CBP-5 (Shimadzu) fused silica capillary column (30 m × 0.25 mm; 0.25 *μ*m film thickness composed of 5% phenylmethylpolysiloxane) and programmed temperature as follows: 60°–240°C at 3°C/min, then to 280°C at 10°C/min, ending with 10 min at 280°C. The carrier gas was He at a flow rate of 1.0 mL/min and the split mode had a ratio of 1 : 20. Compounds were identified by computer search using digital libraries of mass spectral data [19] and by comparison of their retention indices and authentic mass spectra, relative to C8–C32 n-alkane series [20] in a temperature-programmed run.

## 3. Results and Discussion

The yield of *Lippia sidoides* essential oil was 0.8%. Within the identified components of the essential oil, the most abundant were oxygenated monoterpenes, followed by sesquiterpenes hydrocarbons (Table 1).

**Figure 1:**
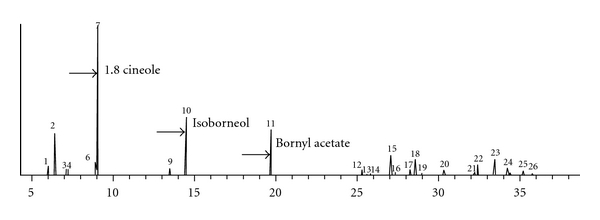
Total ion chromatogram (TIC) of chemical constituents of essential oil from *Lippia sidoides* Cham., (Verbenaceae) leaves, cultivated in Hidrolândia, Goiás, Brazil. (arrow: major constituents).

Altogether, 96.68% of the chemical constituents of the essential oil were identified. As described in Table 1 and by chromatogram showed in Figure 1, 1,8 cineole, an oxygenated monoterpene was mostly constituent (26.67%), followed by isoborneol (14.60%) and bornyl acetate (10.77%).

In *L. sidoides* cultivated in Minas Gerais, Brazil, the 1,8 cineole was also identified in lower concentrations (9.26%) than in this work. However, thymol and carvacrol were also identified [21]. 1,8 cineole was also identified in other species of its kind, such as *Lippia microphylla* Cham. [22]*, Lippia alba* (Mill.) N. E. Brown [6, 23, 24] and *Lippia schomburgkiana* Schauer [24]. 

The results found in this work for *L. sidoides*, cultivated in an area of anthropic bushland, differ from that presented in the literature, where the thymol and carvacrol appear as major components [11–13]. What can be understood when considering that the environment of which the plant develops are factors such as temperature, relative humidity, exposure to the sun and wind, which exert a direct influence on the chemical composition of volatile oils [2, 23, 25]. Alteration in the yield of the essential oils, as well as the quantity of chemical constituents can still be observed due to the different phases of the plant's development [2, 6, 18] and at different times of the year [4, 23]. 

Qualitative and quantitative variations in the composition of the oils can also be observed in species that have chemotypes or chemical races, where botanically identical plants produce different chemical compounds, irrespective of their environment, as registered in *L. alba*, where three chemotypes from different regions and cultivated under the same conditions produce citral, carvone, and linalool, confirming that the variations occur in function of infra-specific variation [6]. Moreover, depending on the liability of the constituents of volatile oils, the method used to extract the essential oils could affect the identification [2, 26]. 

## 4. Conclusion

The chemical composition of essential oil described in this paper differs from that described in the literature for *L. sidoides* found in its native environment, highlighting the need for further studies to assess the variation in chemical composition of vegetal species in different environments, especially those who may have biological activity. 

## Figures and Tables

**Table 1:** Percentage of chemical constituents of essential oil from *Lippia sidoides* Cham. (Verbenaceae) leaves, cultivated in Hidrolândia, Goiás, Brazil.

Constituent	RI	%
Artemisia triene	929	1.71
Camphene	954	6.19
Sabinene	975	1.27
*β*-pinene	979	1.23
*ρ*-cymene	1024	0.38
Sylvestrene	1030	3.03
1,8 cineole	1031	26.67
Cis-sabinene hydrate	1070	0.50
Camphor	1146	1.60
Isoborneol	1160	14.60
Bornyl acetate	1288	10.77
*α*-cedrene	1411	1.75
(e)-caryophyllene	1419	1.09
Cis-thujopsene	1431	1.12
*α*-himachalene	1451	1.37
*α*-humulene	1454	5.66
Ar-curcumene	1480	1.83
*β*-selinene	1490	4.33
Cis-calamenene	1529	2.68
Zierone	1575	1.99
Rosifoliol	1600	4.53
Citronellyl pentanoate	1625	1.79
Alo-himachalol	1662	0.59
Oxygenated monoterpenes	—	53.64
Sesquiterpenes hydrocarbons	—	19.83
Monoterpene hydrocarbons	—	14.31
Oxygenated sesquiterpenes	—	8.90
Unidentified	—	3.32
Total identified (%)	—	96.68

RI: retention indices.
